# The economic burden of stroke: a systematic review of cost of illness studies

**DOI:** 10.25122/jml-2021-0361

**Published:** 2021

**Authors:** Stefan Strilciuc, Diana Alecsandra Grad, Constantin Radu, Diana Chira, Adina Stan, Marius Ungureanu, Adrian Gheorghe, Fior-Dafin Muresanu

**Affiliations:** 1. Department of Neuroscience, Iuliu Hatieganu University of Medicine and Pharmacy, Cluj-Napoca, Romania; 2. RoNeuro Institute for Neurological Research and Diagnostic, Cluj-Napoca, Romania; 3. Department of Public Health, Faculty of Political, Administrative and Communication Sciences, Babes-Bolyai University, Cluj-Napoca, Romania; 4. Center for Health Workforce Research and Policy, Faculty of Political, Administrative and Communication Sciences, Babes-Bolyai University, Cluj-Napoca, Romania; 5. Department of Infectious Disease Epidemiology, Global Health and Development Group, Imperial College London, London, United Kingdom

**Keywords:** stroke, ischemic, hemorrhagic, transient ischemic attack, cost of illness, economic burden

## Abstract

Stroke is one of the leading causes of morbidity and mortality worldwide. As the number of stroke cases is rising from one year to another, policymakers require data on the amount spent on stroke to enforce better financing policies for prevention, hospital care, outpatient rehabilitation services and social services. We aimed to systematically assess the economic burden of stroke at global level. Cost of stroke studies were retrieved from five databases. We retrieved the average cost per patient, where specified, or estimated it using a top-down approach. Resulting costs were grouped in two main categories: per patient per year and per patient lifetime. We extracted information from forty-six cost of illness studies. Per patient per year costs are larger in high income countries and in studies conducted from the payer perspective. The highest average per patient per year cost by country was reported in the United States ($59,900), followed by Sweden ($52,725) and Spain ($41,950). The highest per patient lifetime costs were reported in Australia ($232,100) for all identified definitions of stroke. Existing literature regarding the economic burden of stroke is concentrated in high-income settings, with very few studies conducted in South America and Africa. Published manuscripts on this topic highlight substantial methodological heterogeneity, rendering comparisons difficult or impossible, even within the same country or among studies with similar costing perspectives.

## Introduction

The Global Burden of Disease studies have estimated that in 2017 there were 24.1 million new stroke cases, 15.7 million additional disability-adjusted life years (DALYs) and 700,000 more stroke-related deaths, as compared to the previous year [1, 2]. Both in Europe and the US, stroke has been the leading neurological disease in terms of DALYs [3, 4]. Although stroke cases among young people are rising, stroke is more prevalent in the elderly [5, 6]. As over 9% of the global population is aged 65 or above [7], employing cost of illness studies on stroke will aid health care decision making [8] and help health systems meet, prevent and minimize the strenuous demand of stroke care. The financial burden of stroke on health services and societies is enormous. In Europe, it is estimated that informal care amounted to €1.3 billion, the cost for health care was €27 billion, while the cost due to lost productivity following stroke was €12 billion in 2017 [9]. In the US, the indirect costs amounted to 66% of the total costs ($103.5 billion), with slight differences between the cost of productivity loss ($38.1 billion) and the cost caused by premature death ($30.4 billion) [10].

Cost of illness studies are employed to quantify the economic cost of inpatient, outpatient and other types of care, as well as indirect costs caused by the loss of productivity due to prolonged rehabilitation, temporary or lifelong disabilities, and death. Another cost category is represented by intangible costs, but due to the difficulty in capturing these costs, they are usually not included in the cost of illness studies [11]. As a tool used to estimate the amount spent on a particular disease and as an aid in health financing policy, cost of illness studies are aiming to reconstruct patient pathways (providing comparisons between theoretical and “on-site” pathways), identify relevant stakeholders and cost items and estimate the disease-attributable costs to the society [8]. In addition, cost of illness studies are useful in collecting data on the following cost categories: direct medical costs (mainly attributed to inpatient, outpatient and home care), direct non-medical costs (i.e., due to social services, transportation, childcare) and indirect costs (resulted from productivity losses, cognitive/physical impairments and mortality, among others) [11]. The economic perspective (i.e., societal, provider, patient, or third-party payer) maps the costs components that will be included and quantified in the study, the study scope defines the setting where the study will be conducted (i.e., institutional, regional, national, international) and other study design components such as prospective or retrospective time direction and epidemiological approach (i.e., incidence/prevalence) define the process of data selection and collection [11].

Several systematic reviews focused on the cost of stroke (classified as cardiovascular disease) in patients with type 2 diabetes mellitus [12], hypertension [13] or atrial fibrillation [14]. Other reviews focused exclusively on post-stroke care [15] or stroke-related costs in low and middle-income countries [16]. Some reviews imposed geographical [17] or time-related [18] limitations.

This systematic review aims to compile the results of existing studies on the economic burden of stroke, critically appraise the methodological components and the quality of retrieved studies, fill the existing gaps in the literature and offer guidance for geographical areas lacking scientific outputs on the economics of stroke.

## Material and Methods

Our systematic review was conducted according to PRISMA (Preferred Reporting Items for Systematic Reviews and Meta-Analyses) guidelines [19]. We registered our review protocol (ID CRD42019134654) on PROSPERO (the International prospective register of systematic review). We operated two rounds of protocol amendments during the study, as highlighted by registry entries.

### Search strategy, selection criteria and quality assessment

We used a predefined search strategy containing keywords (“economics”, “costs”, “cost analysis” and “stroke”) and Medical Subject Headings/Emtree terms and we interrogated the following databases to identify relevant studies for our review: PubMed, ScienceDirect, Cochrane Database of Systematic Reviews, Web of Science and EMBASE. Two databases from the original protocol (EconLit and PsycINFO) were excluded from analysis due to inaccessibility during the systematic search. Additional articles were added from systematic reviews using the snowball citation method. The systematic search was performed during July and August 2019.

**Table:** 

**Expanded search strategy**	(“economics”[All Fields] OR “cost”[All Fields] OR “cost analysis”[MeSHTerms] OR (“costs”[All Fields] AND “cost”[All Fields] AND “analysis”[All Fields]) OR “costs and cost analysis”[All Fields]) AND (“stroke”[MeSH Terms] OR “stroke”[All Fields])

Inclusion criteria:

• Primary or secondary data source(s) for stroke-related cost items and their monetary values;
• An observational study design with a cost of illness or economic modeling component (i.e., Markov model);
• A study population comprised of patients over 18 diagnosed with acute ischemic, hemorrhagic, or transient ischemic attack (TIA).

Exclusion criteria:

• Abstracts;
• Grey literature and non-academic studies;
• Studies that were published in a language other than English;
• Studies that reported cost indicators outside the scope of the review;
• Economic evaluations (i.e., cost-effectiveness or cost-benefit analyses);
• Studies not meeting the population inclusion criteria.

No limitations were imposed on the country or date of publication. Two reviewers independently screened study titles, abstracts and the full text of selected articles. Irrelevant studies were removed based on the inclusion and exclusion checklist. Disagreements were generally resolved by consensus and occasionally by a third reviewer. Finally, relevant articles from other systematic reviews retrieved from search results were added to our final selection. Duplicates were removed with OpenRefine data cleaning software (version 3.2).

We evaluated the quality of the included articles using a seven-item checklist derived from the CHEERS (Consolidated Health Economic Evaluation Reporting Standards) checklist that was previously used in a systematic review on the cost of cardiovascular diseases [13]. Of seven questions, five focused on the quality of the economic component and two on the epidemiological component. For analytical purposes, we recorded “yes”, “no” and “unclear” checklist options with numerical values (0, 0.5 and 1), indicating low, medium and high-quality studies, accordingly.

### Data extraction, aggregation and analysis

Descriptive characteristics for each study, such as year, costing methodology, scope, perspective, study design, sample size, country, currency year and country, economic estimate and discount rate, were recorded independently in Microsoft Excel by two analysts. All disagreements were discussed and worked out consensually. The countries where the studies were performed were classified according to income groups proposed by the World Bank [20]. We extracted stroke costs (direct, indirect, or both) from each included study and grouped them across two main indicators: (1) per patient per year and (2) per patient lifetime. When studies reported only total costs and sample sizes, we used a top-down approach to estimate per patient figures by dividing these indicators.

Defining stroke type for result aggregation was difficult due to various clinical (i.e., subtypes and stages of disease) and logistical (e.g., medical coding) factors. To address this issue, we performed preliminary sensitivity analyses to highlight monetary differences across the identified results and to establish aggregation rules based on the major types of stroke: ischemic stroke (IS), hemorrhagic stroke (HS) – encompassing subarachnoid hemorrhage and intracerebral hemorrhage and transient ischemic attack (TIA) [21]. While the case for establishing individual meta-analytic pathways for each subtype of stroke is valid in theory, sub-aggregation is neither feasible (too few studies for individual categories) nor desirable (sensitivity analyses register marginal differences in monetary estimates). Therefore, we chose to present our results based on major stroke types. In eight instances where studies reported several indicators based on these variables, special aggregation rules were applied. Averages were used to aggregate estimates for several institution types (i.e., regional *vs.* county hospital, stroke unit *vs.* non-stroke unit), costing methodologies (i.e., in high *vs.* low prevalence settings), episode of stroke (i.e., first *vs.* recurring stroke) and types of insurance. Sums were used to aggregate stroke subtype cost estimates (i.e., hemorrhagic stroke subtypes).

Costs reported in a currency other than the national one (i.e., cost of illness study performed in China reporting results in USD) were converted back to the national currency. For this, we used either the exchange rate mentioned in the study, if present, or the yearly average rate (retrieved from the National Bank of Italy in the case of Italian Lira and from ofx.com for all other similar cases). If the year of the currency was not reported or could not be determined from the manuscript, it was presumed to be the previous year of the publication date. To adjust identified costs to constant 2020 United States Dollars (USD) values, we used the Campbell and Cochrane Economics Methods Group Evidence for Policy and Practice Information and Coordination Centre (CCEMG – EPPI-Centre) cost converter [22]. The resulting costs were rounded to the nearest hundredth value. Further data analysis was performed based on the extracted costs and descriptive characteristics of the studies. Data curation and analysis were performed using Microsoft Excel, Tableau Desktop (version 2021.1).

## Results

**Figure 1.**
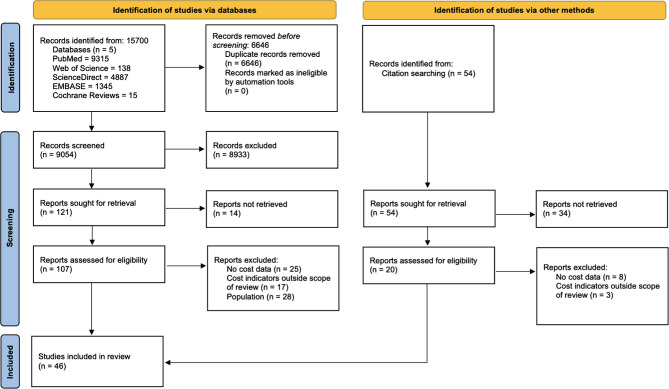
PRISMA Flow Diagram.

Our systematic review retrieved monetary outcomes for various definitions of stroke, including IS, HS and TIA (Figure 1). We included forty-six articles published in twenty-three countries between 1994 and 2019, from which 13% (n=6) were published from 1994 to 2000, 33% (n=15) from 2001 to 2010 and 54% (n=25) from 2011 to 2019. Our final selection included studies estimating the cost of stroke in the following countries: Argentina (n=1), Australia (n=4), Brazil (n=1), Canada (n=3), China (n=3), Denmark (n=2), France (n=2), Germany (n=3), Greece (n=1), Ireland (n=1), Italy (n=3), Korea (n=2), Lebanon (n=1), Malaysia (n=1), Mexico (n=1), Netherlands (n=1), Nigeria (n=1), Pakistan (n=1), Singapore (n=1), South Africa (n=1), Spain (n=2), Sweden (n=4), Tanzania (n=1), Thailand (n=1), Turkey (n=1), United Kingdom (n=2), United States (n=1).

The provider perspective (44%, n=20) and the regional scope (39%, n=18) were the most common methodological features of identified studies (Table 1). Only eight (17%) studies were conducted from the payer perspective. Twenty-five studies (54%) used retrospective data sources to report stroke-related economic outputs. Most of the selected studies did not explicitly report the costing methodology, but used a bottom-up approach to compute costs. Less than a half of the final selection of studies (41%, n=19) included indirect costs and only 17% (n=8) reported direct non-medical costs. Twenty-seven (59%) studies were incidence-based. Eight studies reported applied discounting, with the most common discount rates being 3% and 5%. Fifteen studies (33%) scored below half of the maximum quality score, while nine (20%) were graded with the maximum score of seven on the quality checklist (Table 2).

**Table 1.** Summary characteristics of included studies (n=46).

**Study scope**	**Number of studies – n (%)**
National	11 (24%)
Regional	18 (39%)
Local	4 (9%)
Institutional	12 (26%)
Other	1 (2%)
**Design structure**	
Prospective	21 (46%)
Retrospective	25 (54%)
**Study perspective**	
Payer	8 (17%)
Provider	20 (44%)
Societal	18 (39%)
**Costing methodology**	
Bottom-up	14 (31%)
Top-down	2 (4%)
Both	2 (4%)
Not specified	28 (61%)
**Epidemiological component**	
Incidence	27 (59%)
Prevalence	12 (26%)
Both	7 (15%)

**Table 2.** Quality score of included studies.

**Quality score**	**Number of studies (%)**	**References**
**0**	1 (2)	[23]
**2.5**	3 (6)	[24–26]
**3**	1 (2)	[27]
**3.5**	5 (11)	[28–32]
**4**	5 (11)	[33–37]
**4.5**	4 (9)	[38–41]
**5**	5 (11)	[42–46]
**5.5**	2 (4)	[47, 48]
**6**	6 (13)	[49–54]
**6.5**	5 (11)	[55–59]
**7**	9 (20)	[60–68]

A total of seventy-two cost aggregates were extracted from our selection of studies (Table 3). Most of the costs (92%, n=66) fell into the per patient per year category, with only 8% (n=6) of them being per patient lifetime. 28% (n=20) of the extracted costs were related to identified definitions of stroke, 31% (n=22) were specific for ischemic stroke, 24% (n=17) for hemorrhagic stroke, 6% (n=4) for TIA and 13% (n=9) were costs for mixed types of stroke (i.e., TIA excluded, subarachnoid hemorrhage excluded, or TIA and subarachnoid hemorrhage excluded).

**Table 3.** Cost aggregates extracted from included studies.

**Economic estimate**	**Stroke type (population)**	**Year of publication**	**First author**	**Study perspective**	**Study scope**	**Country**	**Total costs (2020 USD)**
**/Patient/Year**	IS, HS, TIA	1994	Terent et al.	payer	national	Sweden	$81,500
		1995	Martinez et al.	payer	national	Mexico	$38,000
		2000	Claesson et al.	provider	institutional	Sweden	$30,300
		2003	Spieler et al.	provider	regional	France	$32,700
			Youman et al.	societal	institutional	United Kingdom	$60,500
		2004	Rossnagel et al.	societal	local	Germany	$19,200
		2009	Saka et al.	societal	local	United Kingdom	$14,600
		2010	Wei et al.	provider	national	China	$5,600
		2011	Smith et al.	societal	regional	Ireland	$30,200
		2012	Birabi et al.	provider	regional	Nigeria	$5,100
			Lopez-Bastida et al.	societal	regional	Spain	$28,500
		2013	Chevreul et al.	societal	national	France	$17,900
			Kabadi et al.	payer	local	Tanzania	$2,100
		2015	Jennum et al.	societal	national	Denmark	$12,700
			Shuyu Ng et al.	provider	national	Singapore	$11,400
			van Eeden et al.	societal	regional	Netherlands	$39,900
		2016	Alvarez-Sabin et al.	societal	regional	Spain	$45,800
			Maredza et al.	payer	local	South Africa	$2,800
		2018	Abdo et al.	provider	regional	Lebanon	$11,500
	Ischemic stroke	1999	Mamoli et al.	provider	institutional	Italy	$5,500
		2002	Tu et al.	provider	institutional	China	$5,000
		2003	Dewey et al.	societal	regional	Australia	$25,300
			Khealani et al.	provider	institutional	Pakistan	$8,600
		2006	Kolominsky-Rabas et al.	payer	regional	Germany	$29,800
		2008	Gioldasis et al.	provider	institutional	Greece	$6,600
		2009	Christensen et al.	provider	institutional	Argentina	$13,800
		2010	Ma et al.	provider	institutional	China	$2,600
		2011	Asil et al.	provider	regional	Turkey	$3,100
		2012	Mittmann et al.	societal	regional	Canada	$74,200
			Rha et al.	provider	regional	South Korea	$8,300
		2014	Gloede et al.	provider	regional	Australia	$4,600
		2015	Jennum et al.	societal	national	Denmark	$13,500
			Shuyu Ng et al.	provider	national	Singapore	$3,600
		2016	Alvarez-Sabin et al.	societal	regional	Spain	$45,700
			Johnson et al.	payer	regional	United States	$59,900
		2017	Lekander et al.	societal	regional	Sweden	$44,300
		2018	Abdo et al.	provider	regional	Lebanon	$7,000
			Cha	payer	national	South Korea	$11,100
		2019	Safanelli et al.	provider	institutional	Brazil	$8,500
	Hemorrhagic stroke	2003	Dewey et al.	societal	regional	Australia	$27,000
			Weimar et al.	societal	regional	Germany	$53,400
		2008	Gioldasis et al.	provider	institutional	Greece	$11,000
		2009	Christensen et al.	provider	institutional	Argentina	$43,600
		2011	Asil et al.	provider	regional	Turkey	$6,000
		2012	Rha et al.	provider	regional	South Korea	$42,600
		2014	Gloede et al.	provider	regional	Australia	$6,700
			Specogna et al.	provider	other	Canada	$11,000
		2015	Jennum et al.	societal	national	Denmark	$16,300
			Shuyu Ng et al.	provider	national	Singapore	$7,300
		2016	Alvarez-Sabin et al.	societal	regional	Spain	$47,800
		2017	Lekander et al.	societal	regional	Sweden	$54,800
		2018	Abdo et al.	provider	regional	Lebanon	$79,100
			Cha	payer	national	South Korea	$84,900
		2019	Safanelli et al.	provider	institutional	Brazil	$20,600
	Transient ischemic attack	1998	Porsdal & Boysen	payer	institutional	Denmark	$4,000
		2015	Shuyu Ng et al.	provider	national	Singapore	$500
		2018	Abdo et al.	provider	regional	Lebanon	$2,100
		2019	Safanelli et al.	provider	institutional	Brazil	$4,800
	Mixed (TIA excluded)	2012	Nordin et al.	provider	institutional	Malaysia	$3,100
			Rha et al.	provider	regional	South Korea	$12,200
	Mixed (SAH excluded)	1994	Smurawska et al.	provider	institutional	Canada	$35,700
		2001	Dewey et al.	societal	national	Australia	$24,000
		2012	Fattore et al.	societal	national	Italy	$32,200
	Mixed (TIA and SAH excluded)	2005	Gerzeli et al.	societal	national	Italy	$19,900
		2008	Gioldasis et al.	provider	institutional	Greece	$7,500
		2012	Khiaocharoen et al.	provider	regional	Thailand	$3,400
**/Patient Lifetime**	IS, HS, TIA	2016	Zhao et al.	provider	regional	Australia	$232,100
	Ischemic stroke	2014	Ghatnekar et al.	societal	national	Sweden	$75,000
			Gloede et al.	societal	regional	Australia	$60,800
	Hemorrhagic stroke	2014	Ghatnekar et al.	societal	national	Sweden	$104,600
			Gloede et al.	societal	regional	Australia	$48,600
	Mixed (SAH excluded)	2001	Dewey et al.	societal	national	Australia	$56,200

TIA – transient ischemic attack; SAH – subarachnoid hemorrhage.

**Figure 2.**
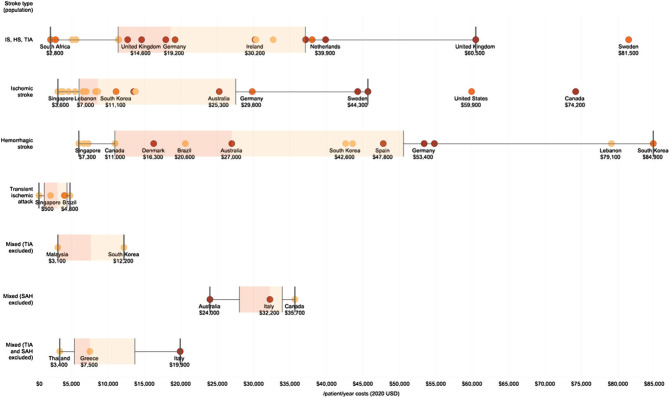
Per patient per year costs (USD 2020) represented as box plots (light orange – provider perspective; orange – payer perspective; dark orange – societal perspective).

**Figure 3.**
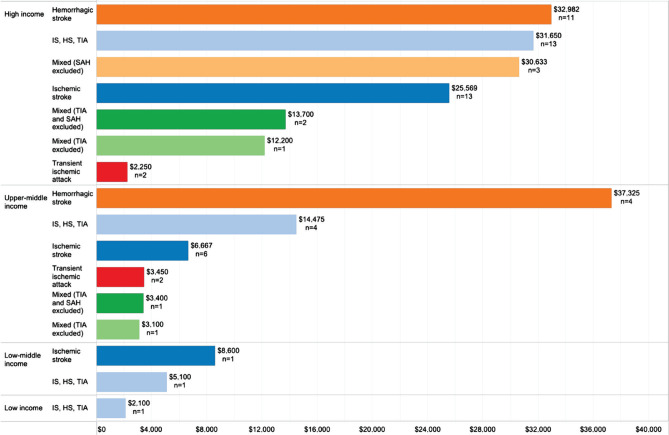
Average per patient per year costs (USD 2020) by country classification and study perspective (n – number of studies).

Costs in the per patient per year category varied from $84,900 in South Korea (for hemorrhagic stroke) to $500 in Singapore (for TIA, however, across all cost perspectives) (Figure 2). Average per patient per year costs is greater in high-income countries and in studies conducted from the payer perspective (Figure 3).

**Figure 4.**
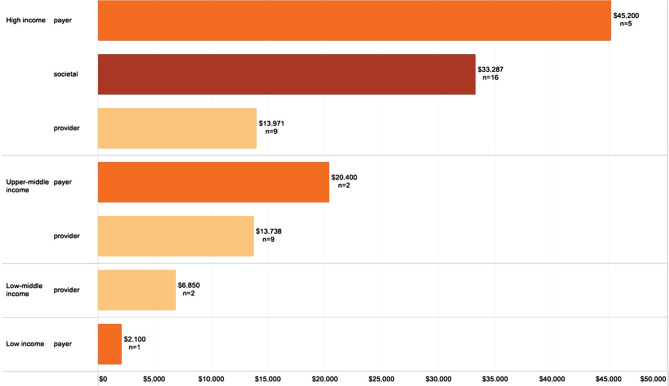
Average per patient per year costs (USD 2020) by country classification and study perspective (light orange – provider perspective; orange – payer perspective; dark orange – societal perspective) (n – number of studies).

Hemorrhagic strokes are the costliest type of stroke in both high-income and upper-middle-income countries, as represented in Figure 4.

**Figure 5.**
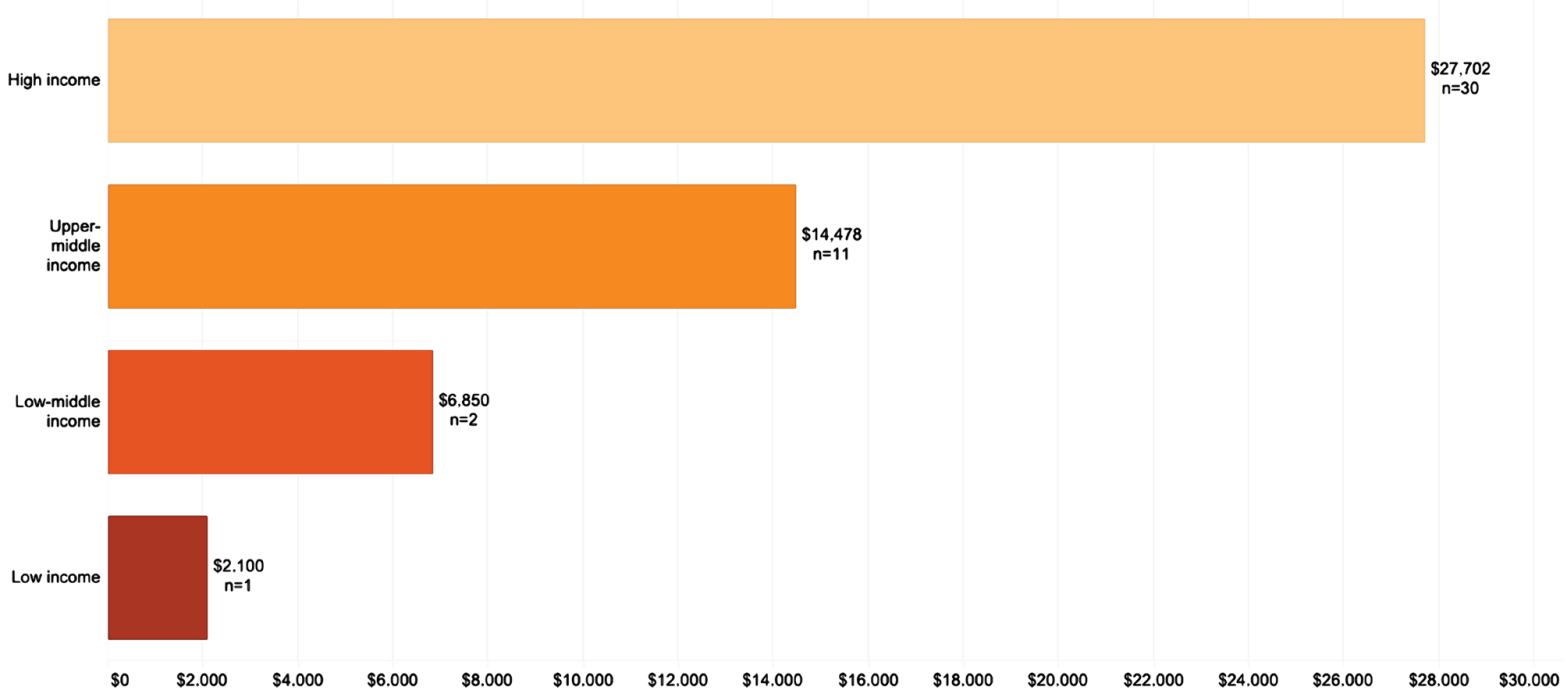
Per patient per year costs (USD 2020) by country classification (n – number of studies).

**Figure 6.**
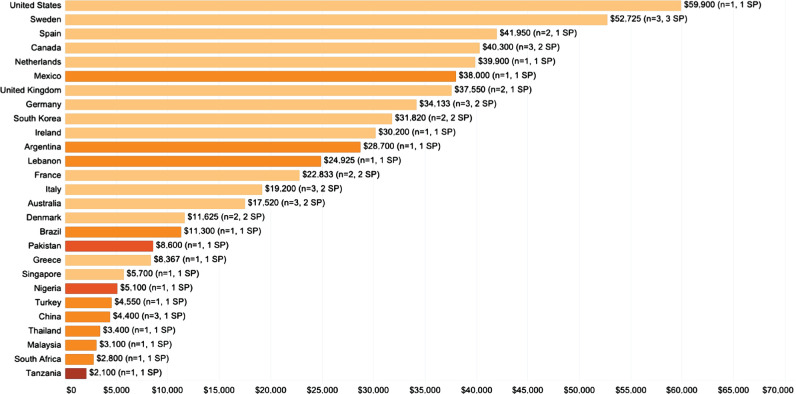
Cost estimates aggregated by country. Colors represent World Bank income classification (light orange – high income; orange – upper-middle-income; dark orange – low-middle income; brown – low income; SP – study perspectives used in the analyses; n – number of studies).

**Figure 7.**
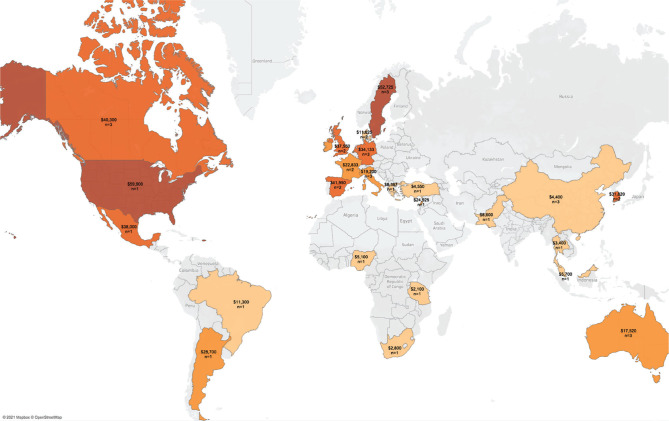
World map reflecting per patient per year costs (USD 2020). Map figures show country averages across studies analyzed (color gradient: light – lower values, dark – higher values; n – number of studies).

The mean per patient per year cost of stroke in high-income countries was $27,702, while for upper-middle-income countries, it was $14,478 (Figure 5). The highest average per patient per year cost by country was registered in the United States ($59,900), followed by Sweden ($52,725) and Spain ($41,950) (Figures 6, 7).

**Figure 8.**
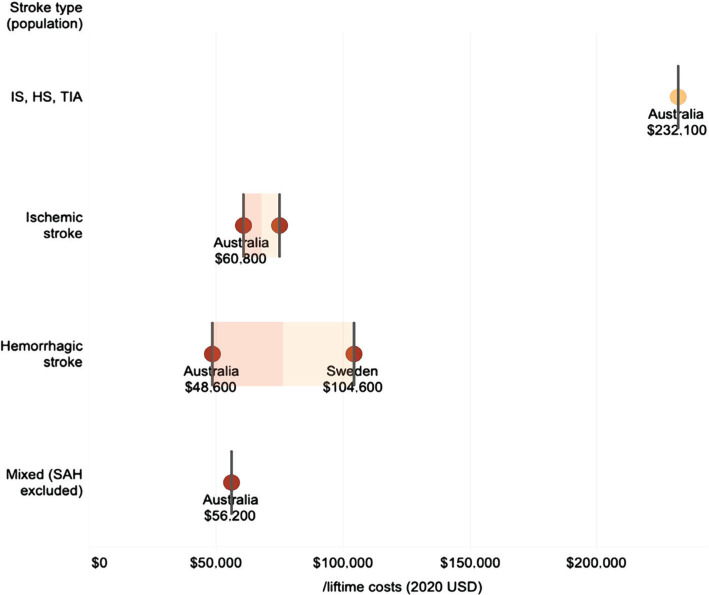
Per patient lifetime costs (USD 2020) represented as box plots (light orange – provider perspective; dark orange – societal perspective).

As for lifetime costs, the highest was registered in Australia ($232,100) for IS, HS and TIA, in a study conducted from the provider perspective. The average lifetime costs for hemorrhagic stroke are slightly higher than those for ischemic stroke ($75,600 *vs.* $67,900) (Figure 8).

## Discussion

This systematic review aimed to critically assess and summarize existing literature on the economic burden of stroke, using a broad search strategy that encompasses several disease subtypes, economic perspectives and study scopes. Existing literature regarding the economic burden of stroke is concentrated in high-income settings, with very few studies conducted in South America and Africa. Published manuscripts on this topic highlight substantial methodological heterogeneity, rendering comparisons difficult or impossible, even within the same country or among studies with similar costing perspectives. Very few manuscripts report information on transient ischemic attacks.

The most prominent evidence gap we have observed in the literature is the geographical one. As illustrated by Figures 6 and 7, most cost of stroke studies are concentrated in Europe and North America. There are very few countries in Asia, Africa, Eastern Europe and South America where such studies have been performed, rendering the endeavor of forming a global perspective on the economic burden of stroke highly difficult. Moreover, the existing body of literature originates predominantly from high and upper-middle-income countries. As such, conclusions based on this data may be skewed by the demographic characteristics, economic environment and the maturity of these countries’ health systems. For similar reasons, drawing appropriate national-level comparisons and conclusions regarding the economic burden of stroke is an equally daunting task. Based on our results, all countries with two or more eligible studies rendered by our systematic search report diverging monetary estimates, indicating a high level of output uncertainty.

There is a high degree of variation between the methodologies employed by the studies we analyzed. Most authors did not specify the costing methodology used. As for the indicators used, the overwhelming majority of cost aggregates identified in the studies cover a single year in the life of a stroke patient (most often the first year after the event), while only a handful address lifetime costs.

In addition, very few papers quantify indirect or direct non-medical costs and even fewer are focused on TIAs. As for the economic perspectives from which the studies have been performed, the dominant one is the provider perspective. The approval and implementation of intravenous treatment with rtPA [69, 70], access to stroke units [71], issued guidelines on hospital care management [72], primary [73] and secondary stroke prevention [74, 75], have all contributed to reducing the burden of stroke and its mortality and have increased the number of survivors [76]. Stroke survivors are affected by a wide range of temporary or long-term physical and cognitive impairments [77–80], which require inpatient and outpatient neurorehabilitation interventions, with pharmacological, physical and psychological components tailored for each recovery phase [81–84]. It would be therefore essential that future studies address as many dimensions of the economic burden of stroke as possible.

With the increasing prevalence of stroke cases [85], when designing cost of stroke studies, loss of productivity, neurorehabilitation and secondary prevention need to be considered in order to properly quantify costs for prevention, tertiary health providers (stroke ranks third among neurological disorders requiring the highest need of rehabilitation [86]), employers (as stroke survivors aged over 60 with low levels of education and diabetes were more likely to be unemployed [87]) and social services. Because stroke survivors require aid in carrying out daily activities and sometimes need constant supervision [88], informal care is another component that needs to be considered in the study design.

Particularly in underperforming health systems, limited access to data is one of the most important barriers to developing cost of illness studies. While electronic medical records or claims databases enable an analysis of various clinical details and the cost incurred by stroke patients during their hospitalization, these are not available or appropriately developed in many countries. Retrospective cost information may also be extracted from national stroke registries, hospital records, or secondary data from more extensive projects focusing on collecting data on the incidence [89], costs [49] and quality of life in stroke patients [90]. These are some advantages of using retrospective data, such as reduced study costs and time (as the data has already been collected) and the possibility of stratifying patients according to age, sex, severity, stroke recurrence, geographical indicators (urban/rural, city, regional and national level), or other stroke classification systems used in clinical settings (such as the OCSP – Oxfordshire Community Stroke Project classification [91] and TOAST – Trial of Org 10172 in Acute Stroke Treatment [92]). However, secondary data sources have several disadvantages. For example, additional costing and clinical data may not be linked and important cost categories may not be included (such as health services provided in a different hospital or primary care, as well as out-of-pocket payments for medicines [93], additional rehabilitation services provided at home, or assistance from a hired caregiver). As for the quality of hospital data, it can present errors [94]. Prospective studies allow control over variable selection yet are more consuming in terms of resources. They present challenges due to loss to follow-up [95] and because data collection might be affected by not recalling all relevant costs or by limited availability of complete data when using the diary method.

We did not analyze specific cost items of direct and indirect costs due to study heterogeneity. As mentioned in our methods section, a language restriction was imposed, thus limiting our study results. We have also omitted grey literature such as figures provided by professional societies due to diverging methodological approaches that would risk adding further bias to our conclusions. On the other hand, these limitations are balanced by the broadness of our approach (i.e., providing an overview of studies published worldwide in an unrestricted time window, focusing on all stroke categories, perspectives, scopes, costing methodologies and epidemiological approaches).

Overall, a much wider coverage and a more homogenous methodological approach are necessary to draw overarching conclusions regarding the full economic impact of stroke across countries and continents. Accurate cost estimates at each point in the stroke continuum of care, considered from a broader societal perspective, are essential for better stroke-related financing policy. The findings highlighted by our manuscript have several implications for optimizing stroke care. First, a significant number of countries have no access to any type of information of this kind. While international figures may be extrapolated or otherwise used to develop estimates for such cases, it is crucial to note that in the case of such a complex affliction, patient pathways inevitably diverge based on clinical guidelines, existing infrastructure and resources allocated for health. Hence, developing evidence to support the specific needs of a health system is probably a worthwhile investment for policymakers seeking to improve standard of care. Secondly, where evidence exists, large variations in monetary figures and methodological approaches not only render aggregation and comparisons difficult but introduce uncertainty in the policymaking process. In some cases, this lack of consensus and clarity regarding economic estimates within a country may empower the use of circumstantial evidence to drive decision-making (e.g., alternating use of estimates based on particular policy agendas). Conversely, extrapolating or transferring cost estimates from other countries, however similar, should be conducted, when appropriate, with great caution and care for understanding how the cost estimates were produced.

## Conclusion

As the prevalence of stroke among the active population increases, studies with broader societal perspectives and harmonized protocols could significantly improve health system resource allocation. Based on our experience with synthesizing existing evidence on the economic burden of stroke for this systematic review, we assert that establishing a standardized, internationally agreed framework for future costing exercises focused on stroke is very much needed for several reasons. First, such aggregation efforts should enable robust comparisons across countries, rendering recommendations for health system research allocation. Moreover, based on common (gold) standards of care and similar patient pathways, costing benchmarks for similar services could be established to promote efficient use of resources. Most notably, reducing methodological heterogeneity is crucial to avoid introducing aggregation bias due to diverging study perspectives, scopes and structural parameter uncertainty. Nevertheless, a common costing framework should not discourage the use of various perspectives, including patient, caregiver, government and societal ones.

## Acknowledgements

### Conflict of interest

The authors declare that there is no conflict of interest.

### Authorship contributions

SS, DAG, DC: conceptualization and methodology. SS, DAG, DC and CR: investigation, data curation, formal analysis and vizualisation. AS, MU, AG and DFM: supervision and validation. All authors contributed to writing the original draft, reviewing and editing the manuscript.
